# Evolution of the Free Energy Landscapes of *n*-Alkane Guests Bound within Supramolecular Complexes

**DOI:** 10.1021/acs.jpcb.1c03640

**Published:** 2021-06-25

**Authors:** Busayo D. Alagbe, Bruce C. Gibb, Henry S. Ashbaugh

**Affiliations:** † Department of Chemical and Biomolecular Engineering, Tulane University, New Orleans, Louisiana 70118, United States; ‡ Department of Chemistry, Tulane University, New Orleans, Louisiana 70118, United States

## Abstract

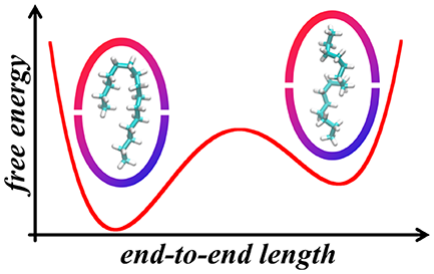

Confinement within nanoscale spaces can dramatically alter the ensemble of conformations flexible species explore. For example, chaperone complexes take advantage of confinement to fold misfolded proteins, while viral capsids transport genomic materials in tight packings. Here we examine the free energy landscapes of *n*-alkanes confined within supramolecular dimeric complexes of deep-cavity cavitand octa-acid, which have been experimentally demonstrated to force these chains with increasing length to adopt *extended*, *helical*, *hairpin*, and *spinning top* conformational motifs, using molecular simulations. Alkanes up to *n*-docosane in both vacuum and water predominantly exhibit a free energy minimum for elongated conformations with a majority of *trans* dihedrals. Within harmonically sealed cavitand dimers, however, the free energy landscapes as a function of the end-to-end distance between their terminal methyl units exhibit minima that evolve with the length of the alkane. Distinct free energy basins are observed between the *helical* and *hairpin* motifs and between the *hairpin* and *chicane* motifs whose relative stability changes with the number of carbons in the bound guest. These changes are reminiscent of two state-like protein folding, although the observed alkane conformations confined are more insensitive to temperature perturbation than proteins are. While the *chicane* motif within the harmonically sealed dimers has not been observed experimentally, this conformation relaxes to the observed *spinning top* motif once the harmonic restraints are released for the complexes in aqueous solution, indicating that these motifs are related to one another. We do not observe distinct minima between the confined *extended* and *helical* motifs, suggesting these conformers are part of a larger *linear* motif family whose population of *gauche* dihedral angles grows in proportion to the number of carbons in the chain to ultimately form a helix that fits the alkane within the complex.

## Introduction

**Figure 1:**
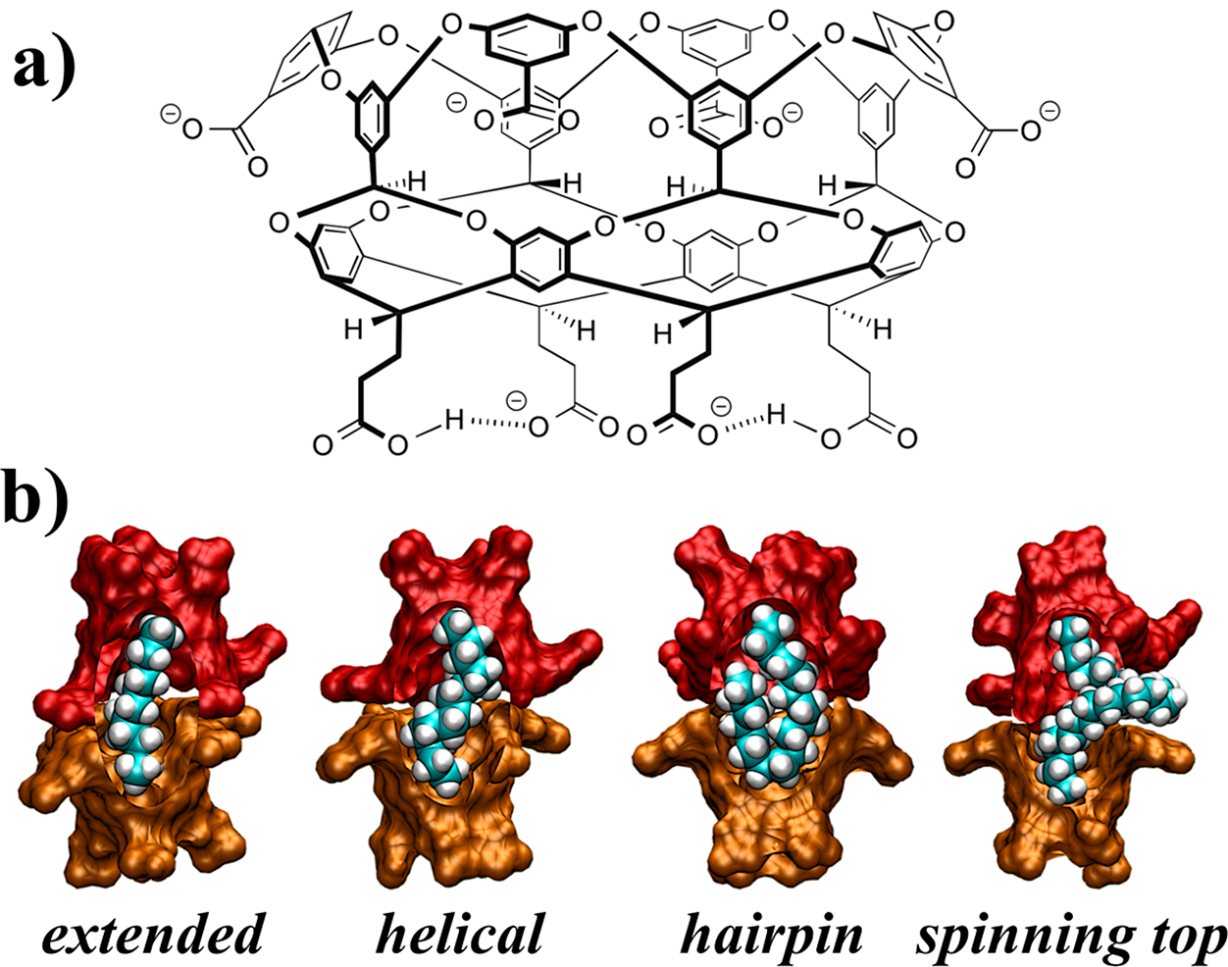
(**a**) Chemical structure of deep-cavity cavitand octa-acid. At pH 7 the carboxylic acids ringing the rim of the hydrophobic pocket at the top of the cavitand are deprotonated and carry a negative charge (−), while only two of the carboxylic acids of the four feet at the bottom of the cavitand are deprotonated. (**b**) Simulation snapshots of alkanes contained within a cavitand dimer complex exhibiting an *extended* (C_11_), *helical* (C_14_), *hairpin* (C_18_), or *spinning top* (C_22_) conformational motif. The alkanes are illustrated as a teal (carbon) and white (hydrogen) van der Waals surface, while the two cavitands are illustrated as the red or orange solvent accessible surfaces. Part of the cavitand dimer structure has been peeled away to show the alkane buried within each complex.

The spontaneous organization of molecular host and guest species in solution into well-defined supramolecular assemblies offers an attractive route toward building synthetic complexes that potentially possess biomimetic functions. Indeed, organisms utilize nanoscale compartmentalization to enable processes like catalysis, transport, storage, and protection. The GroEL/GroES^1,2^ and CCT/TRiC^3−5^ complexes, barrel-like assemblies of chaperonin proteins, internalize misfolded proteins and facilitate their refolding to their native structures to prevent the buildup of toxic protein aggregates. Virus capsids, on the other hand, are large supramolecular protein complexes that serve as transport containers to traffic viral RNA/DNA under pressure between organisms.^6−8^ In 2004, Gibb and co-workers synthesized deep-cavity cavitand octa-acid (OA), a host molecule possessing three rows of aromatic rings that compose a hydrophobic pocket approximately 8 Å in diameter and 8 Å deep (Figure 1a).^9,10^ The rim and foot of this pocket are functionalized with eight carboxylic acid coating groups that engender aqueous solubility. The nonpolar pockets of OA readily bind to hydrophobic guest species in water to form 1:1, 2:1, and 2:2 supramolecular complexes (denoted *X*:*Y*, where *X* and *Y* are the number of host/cavitand and guest species in a complex) depending on the guest’s size and shape.^11−13^ Guests confined within these supramolecular complexes can adopt conformations rarely observed in bulk solution, which in turn can enable the cavitand to mimic an enzymatic pocket.^14,15^ Encapsulation of α-alkyl dibenzyl ketones within 2:1 complexes with OA, for example, was found to yield distinct photoreaction products depending on the packing of constraints imposed by packing of the alkyl side chain within the nonpolar interior.^16^ Cyclization of α,ω-thio-alkane halides within cavitand complexes, on the other hand, has been shown to be promoted by both templation by the host for the guest to adopt a *J*-like/*hairpin* conformation and the charge (anionic or cationic) of the host complex to lower the barrier for creating the negatively charged transition species.^17,18^ Experimental analysis of the packing of *n*-alkanes within 2:1 complexes with OA using one- and two-dimensional NMR found these guests exhibited a succession of conformational motifs with increasing chain length (Figure 1b). Beginning with *n*-nonane, the shortest guest to stabilize 2:1 complexes, the guest adopted an *extended* motif enriched in *trans* dihedrals down its backbone. The inherent spatial limitations inside the complex compressed alkanes of increasing length, forcing the population of *gauche* dihedrals to increase so that alkanes like *n*-tetradecane were found to adopt a *helical* motif. Around *n*-heptadecane the strain imposed by the growing *gauche* population forced the guest to adopt a *hairpin* motif, in which the two methyl termini are directed toward a single OA host pocket while the hairpin turn is directed toward the opposing pocket in the dimer. Ultimately, guests longer than tricosane were found to adopt a *spinning top* motif where the terminal methyl units are redirected toward the opposing host pockets while the hairpin turn is extruded between the two hosts and partially exposed to the surrounding aqueous environment. While the interiors of the cavitand dimers are dry, the capacity for water to enter into such confined spaces is anticipated to play a role in determining the conformation of encapsulated alkanes as well.^19^

Over the past 10 years Ashbaugh and Gibb have examined the stability and function of cavitand complexes in water in a joint experimental and simulation collaboration.^20^ In one of our first studies, we demonstrated that molecular simulations accurately reproduce the succession of alkane packing motifs inferred from NMR as a function of the guest length.^21^ The bound alkane motifs were identified by visual inspection of the most probable conformation over a simulation trajectory for each guest obtained by dihedral principal component analysis.^22−26^ Gauge invariant atomic orbital^27^ calculations performed on the most probable bound alkane conformation from simulation yielded semiquantitively accurate predictions of the experimental shifts in the one-dimensional NMR chemical shifts of the bound guest protons providing confidence in the accuracy of our simulation results. Free energies associated with growing the alkanes within OA dimers by addition of −CH_2_– groups found breaks in the incremental free energy change at the chain lengths at which the dominant motif changed. While this suggested that the conformational motifs observed were associated with transitions between distinct basins in the underlying free energy landscape, we could not directly address the thermodynamic differences between conformers. Characterization of the free energy landscapes of free and bound guests within supramolecular complexes would provide valuable insight into the impact of confinement on orchestration of the guest conformation.

Here we revisit our earlier simulation study of *n*-alkane conformations within cavitand host dimers to examine the evolution of guest free energy landscapes under confinement. Molecular dynamics (MD) simulations are performed to analyze the potentials-of-mean force (PMFs) between the terminal methyl units of the alkanes *n*-undecane to *n*-docosane in vacuum, in water, and confined within harmonically sealed cavitand complexes. The changes in the PMFs with increasing guest length subsequently enable characterization of the impact of confinement on the conformational landscape of the chains, determination of the equilibrium conformational motifs exhibited by the confined chains, and examination of the changes in the PMFs with increasing guest length to determine the thermodynamic driving forces determining the dominant conformational motif. Finally, we release the harmonic restraint between the cavitands to unseal the dimer complexes in aqueous solution. This allows the dimer capsule some latitude to “breathe” and hence reveal the equilibrium guest conformation that would be experimentally observable.

## Simulation Methods

MD simulations of *n*-alkanes and OA dimeric capsules were performed using the GROMACS 2016.3^28^ simulation package. Four sets of simulations were carried out: *n*-alkanes in a vacuum; alkanes in bulk water; alkanes confined within a harmonically sealed OA dimer in vacuum; and alkanes within an unsealed OA dimer in water. The alkanes (denoted C_*n*_, were *n* is the number of carbons in the alkane chain) considered ranged in length from undecane (C_11_) to docosane (C_22_). The alkanes were modeled using the L-OPLS all-atom force field.^29^ The OA cavitands were modeled using the generalized Amber force field (GAFF)^30^ with partial charges obtained from AM1-BCC calculations.^31^ The net charge of each cavitand was set to −6e to match the expected protonation state at pH 7.^32^ To achieve this charge state, the four acidic coating groups ringing the hydrophobic pocket at the top of OA and two acids diagonal to one another at the foot of OA were deprotonated (Figure 1a). Anionic charges on OA were neutralized by 12 sodium cations (6 per OA) modeled using GAFF. Water was modeled using the TIP4P-Ew potential.^33^ Bonds involving hydrogens for the hosts and guests were constrained using the LINCS algorithm,^34^ while water was held rigid using SETTLE.^35^ The equations of motion were integrated using a leapfrog algorithm with a time step of 2 fs.

In the first set of simulations, individual alkanes were placed in vacuum to determine the potential of mean force (PMF) as a function of the end-to-end distance (*r*_ee_) between the terminal methyl end groups of the chain. The alkanes C_12_, C_13_, C_15_, C_17_, C_18_, C_19_, C_21_, and C_22_ were considered here, while determination of the PMFs of the remaining chains in vacuum is described immediately below. Simulations were conducted at 25 °C in the canonical ensemble. The temperature was controlled using the Nosé–Hoover thermostat.^36,37^ No potential truncation or periodic boundary conditions were applied in the vacuum. The end-to-end PMFs were determined using umbrella sampling^38^ over a series of overlapping windows from the terminal methyl units in contact to the fully elongated chain. Overlapping windows (30–55) were used depending on the alkane length. The harmonic umbrella potential utilized a force constant of 50 kJ/(mol Å^2^) with successive minima evenly spaced in 0.5 Å increments. Each window was simulated for 10 ns for sampling following 2 ns of equilibration. The PMFs were reconstructed using the weighted histogram analysis method (WHAM).^39^

In the case of C_11_, C_14_, C_16_, and C_20_, we performed replica exchange molecular dynamics (REMD) simulations to determine their vacuum PMFs as a function of temperature so that the enthalpic and entropic contributions to the free energy could be determined. Simulations were conducted for a single chain in vacuum in the canonical ensemble with the temperature maintained using the Parrinello velocity rescaling algorithm.^40^ Ten replicas were considered at temperatures ranging from 25 to 226.85 °C (298.15, 317.79, 338.46, 360.20, 383.13, 407.22, 432.55, 459.21, 487.29, and 500 K). These temperatures were assigned using the Patriksson and van der Spoel^41^ algorithm to ensure maximal exchanges between neighboring temperatures. An exchange rate of 55% was obtained. Following equilibration, the end-to-end PMF between the alkane methyl groups was determined using umbrella sampling in combination with REMD. Here, 50–80 overlapping windows determined by the alkane length were simulated with a harmonic umbrella potential utilizing a force constant of 50 kJ/(mol Å^2^) with successive minima evenly spaced in 0.32 Å increments. Equilibration runs were conducted for 4 ns, after which each window was simulated for 20 ns to gather statistics.

In the second set of simulations, an individual alkane (C_11_–C_22_) was placed in a bath of 5000 water molecules to determine the end-to-end PMF of the alkane. Simulations were conducted in the isothermal–isobaric ensemble at 25 °C and 1 bar. In addition to using the Nosé–Hoover thermostat, the pressure was maintained using the Parrinello–Rahman barostat.^42^ Nonbonded Lennard-Jones interactions were truncated beyond a separation of 9 Å with a mean-field dispersion correction for longer-range contributions to the energy and pressure. Electrostatic interactions were evaluated using particle mesh Ewald summation with a real space cutoff of 9 Å.^43^ The PMF was evaluated using the same protocol and number of windows as our first set of simulations of alkanes in vacuum.

In the third set of simulations, we examined the end-to-end PMFs of alkanes confined within a cavitand dimer in vacuum. The presumption is that the chain conformation under confinement in vacuum is comparable to that in solution, that is the solvent plays only a secondary role in the chain conformation. To ensure the 2:1 supramolecular complexes did not disassemble in vacuum, harmonic restraints were applied between eight pairs of carbons ringing the mouths of the cavitands to seal the dimer.^21^ The harmonic bond length and spring constant used to seal the dimer were 4 Å and 25 kJ/(mol Å^2^), respectively. These simulations were conducted in the canonical ensemble with the temperature maintained using the Parrinello velocity rescaling algorithm.^40^ Given that the chains are conformationally constrained within the dimer, initial equilibration of the alkanes to relax the chain conformation was conducted using REMD.^44^ Pre-equilibration was conducted for 100 ns over 10 temperatures from 25 to 226.85 °C (298.15, 317.79, 338.46, 360.20, 383.13, 407.22, 432.55, 459.21, 487.29, and 500 K). These temperatures correspond to the same temperatures used in the vacuum REMD simulations above. An exchange rate of 40% was obtained. Following equilibration, the end-to-end PMF between alkane methyl groups was determined using umbrella sampling in combination with REMD. Fifty overlapping windows were simulated with a harmonic umbrella potential utilizing a force constant of 50 kJ/(mol Å^2^) with successive minima evenly spaced in 0.32 Å increments. The final configurations of the pre-equilibration runs were used as the initial configuration of the secondary equilibration run with the umbrella potential applied. The secondary equilibration runs were conducted for 4 ns. Following secondary equilibration, each window was simulated for 20 ns to gather statistics for the PMF. The PMFs were reconstructed using WHAM. Here 10 000 configurations of each window were saved for dihedral principal component analysis (*vide infra*) to determine the dominant chain conformation within the complex for specified windows.

In the final set of simulations, we considered the unsealed host–guest complex in aqueous solution. The initial guest configuration for these simulations were taken from the final configuration of the dimers in vacuum above whose umbrella potential minimum was closest to the minimum in the end-to-end PMF of the alkane. The harmonic restraints sealing the dimer were released and the complex was placed in a bath of 2500 waters. These simulations were conducted at 25 °C and 1 bar pressure using the Nosé–Hoover thermostat and Parrinello–Rahman barostat, respectively. Following equilibration of 4 ns, these simulations were conducted for 100 ns. Also, 50 000 configurations were saved for dihedral principal component analysis (*vide infra*) to determine the dominant chain conformation within the complex.

The REMD simulations described above provided PMFs over a wide temperature range from which the enthalpies and entropies of interaction can be evaluated. Specifically, we fit the simulation PMFs to the function
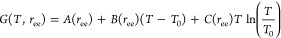
1where *G*(*T*, *r*_ee_) is the temperature dependent PMF as a function of *r*_ee_, *A*(*r*_ee_), *B*(*r*_ee_), and *C*(*r*_ee_) are temperature independent functions of *r*_ee_, and *T*_0_ is a reference temperature assumed here to be 298.15 K. This function effectively assumes the interaction heat capacity is independent of temperature. While higher order terms could be included in this expression to incorporate a temperature dependence for the heat capacity, the fits obtained were not statistically improved. The temperature independent coefficients were obtained by least-squares fitting of eq 1 to the simulation PMFs. The enthalpy and entropy are obtained from the temperature derivatives of eq 1
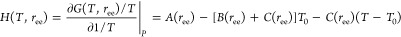
2aand
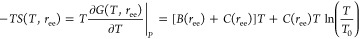
2bAt *T*_0_ the free energy, enthalpy, and entropy are *G*(*T*_0_,*r*_ee_) = *A*(*r*_ee_), *H*(*T*_0_, *r*_ee_) = *A*(*r*_ee_) – [*B*(*r*_ee_) + *C*(*r*_ee_)]*T*_0_, and −*T*_0_*S*(*T*_0_, *r*_ee_) = [*B*(*r*_ee_) + *C*(*r*_ee_)] *T*_0_, respectively.

The dominant conformation of the encapsulated *n*-alkanes was obtained from dihedral principal component analysis (DPCA)^22,23,25,26^ which has previously been applied to examine the structures of proteins in bulk solution and *n*-alkanes in water^45^ and confined within cavitand dimers.^21^ To determine the dominant alkane conformation from a given set of simulation configurations, DPCA was used to establish the two dominant eigenvectors of the dihedral covariance matrix exhibiting the greatest variance during the simulations. A probability distribution of chain configurations mapped to the eigenvector coordinate system was subsequently obtained. From this probability distribution, the simulation alkane configuration that fell closest to the maximum in probability was subsequently judged to be the dominant chain configuration. For the simulations of the dimers in vacuum this analysis was conducted over the specified window containing the local free energy minima in the end-to-end PMFs of the alkanes, while for the simulations of the dimers in water the analysis was conducted over the entire simulation trajectory.

## Results and Discussion

**Figure 2:**
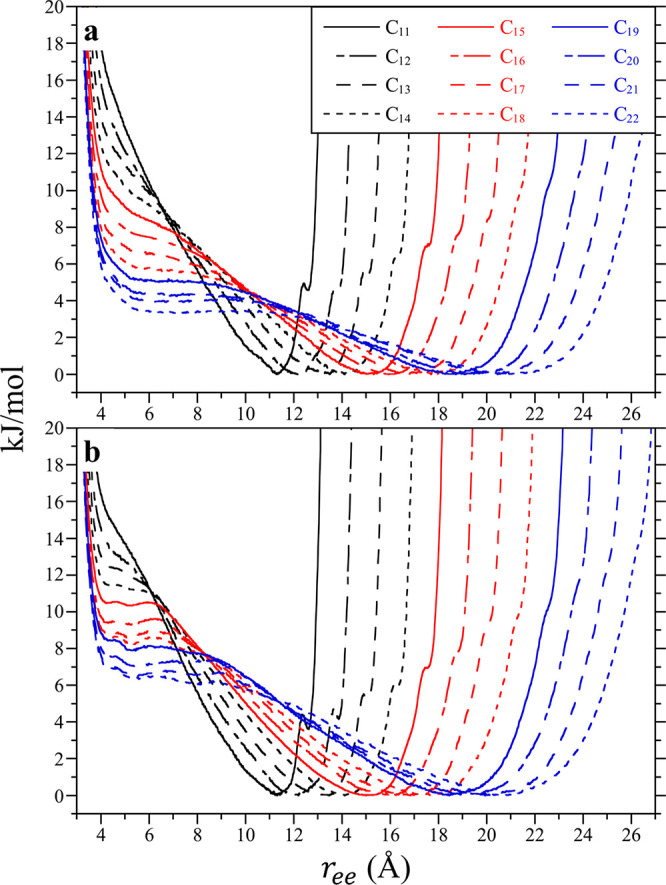
Potentials-of-mean force between the terminal methyl units of the alkanes (C_11_–C_22_) as a function of their end-to-end separation (*r*_ee_) at 25 °C in vacuum (**a**) and water (**b**). The figure lines for each alkane are defined in the legend of **a**. Error bars are neglected for clarity.

The end-to-end PMFs between the terminal methyl units of the alkanes C_11_–C_22_ in vacuum and water at 25 °C are reported in Figure 2. In vacuum (Figure 2a), the PMF for C_11_ exhibits a dominant free energy minimum at an end-to-end separation (*r*_ee_) of 11.3 Å. This separation is slightly less than expected if the chain was in an all-*trans* conformation. We observe a secondary PMF minimum at a higher free energy at *r*_ee_ = 12.6 Å corresponding to the all-*trans* conformation. As discussed below, the primary minimum occurring at 11.3 Å is a result of the entropic need of the alkane to incorporate a minority population of *gauche* dihedrals down the chain’s backbone despite the fact the all-*trans* conformation has the lowest intramolecular energy. Unsurprisingly, increasing the alkane length shifts the primary minimum in the end-to-end PMF in vacuum to more distant separations. Concurrently, the secondary, all-*trans* minimum becomes even less favorable (i.e., higher free energy) and less prominent. In addition, the free energy at a ring closing end-to-end separation of ∼5 Å becomes lower and more favorable with increasing size, such that for C_22_ a shallow secondary minimum is even observed. The free energy of the extended chain is still lower than that of the ring closing state, so that C_22_ is expected to be elongated in vacuum.

**Figure 3:**
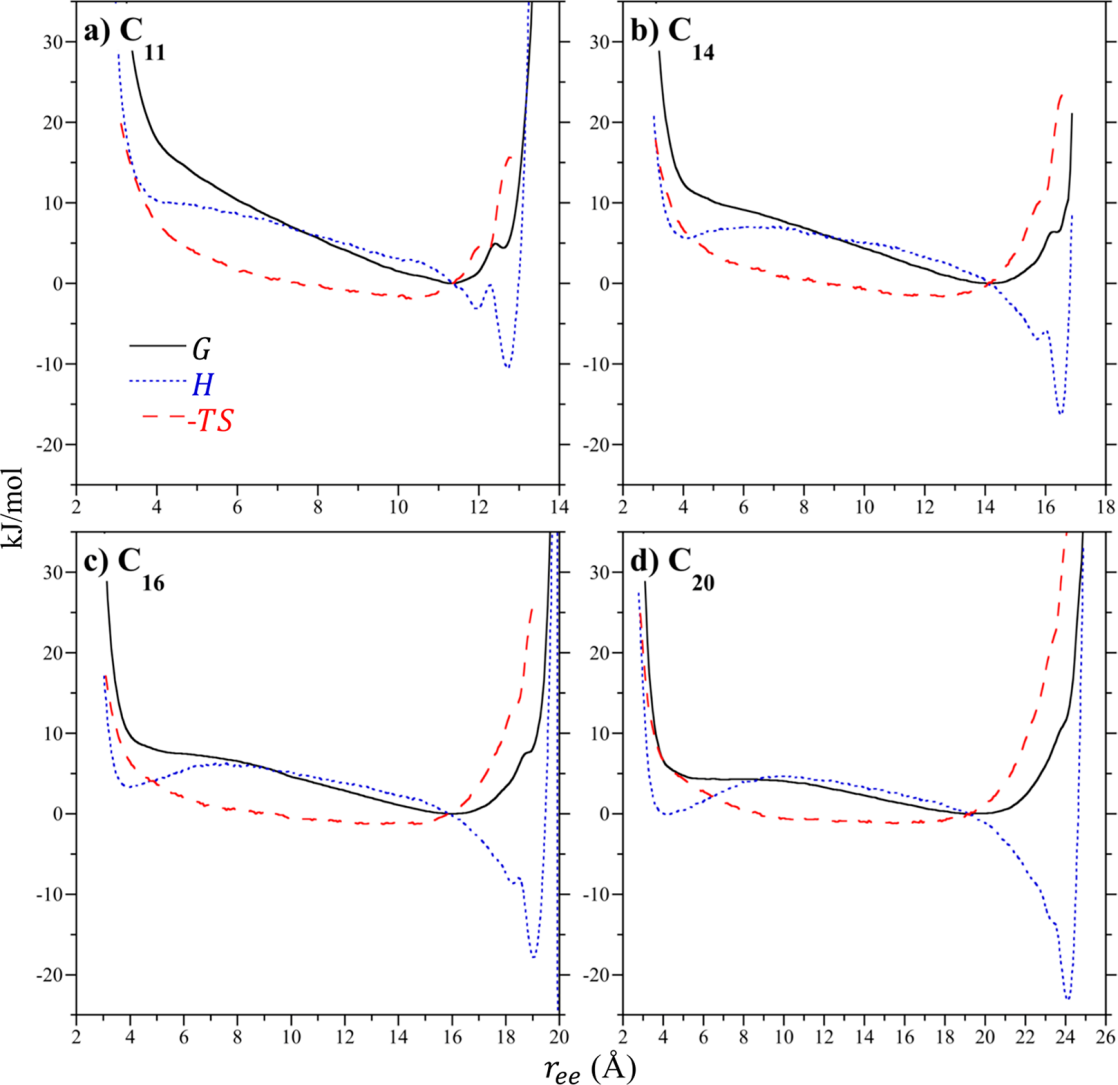
Decomposition of the alkane terminal methyl unit potentials of mean force into their enthalpic and entropic components for alkanes in a vacuum at 25 °C. Results as reported for (**a**) C_11_, (**b**) C_14_, (**c**) C_16_, and (**d**) C_20_. The lines for the enthalpic (*H*) and entropic (−*TS*) contributions to the free energy/PMF (*G*) are defined in the legend of **a**.

The vacuum PMFs of C_11_, C_14_, C_16_, and C_20_ are broken down into their enthalpic and entropic contributions at 25 °C in Figure 3. In difference to the PMF, the minimum in the end-to-end enthalpy for C_11_ occurs for the all-*trans* chain (Figure 3a). This energetic minimum of −10 kJ/mol is significantly deeper that the free energy minimum (normalized to 0 kJ/mol), amounting to ∼4*RT*. This enthalpy minimum is opposed, however, by an even more unfavorable entropy for the chain to adopt the all-*trans* conformation. This repulsive entropic contribution almost certainly arises from the fact that there is only one way for the chain to adopt an all-*trans* conformation while there are considerably more ways to adopt less extended conformations. Indeed, the minimum in the end-to-end entropy of C_11_ occurs at *r*_ee_ = 10.5 Å, shorter than the free energy minimum at 11.3 Å. Below the end-to-end separation of 10.5 Å, both the enthalpy and the entropy of C_11_ become increasingly unfavorable. The enthalpies and entropies of C_14_, C_16_, and C_20_ are qualitatively similar to that of C_11_ (Figures 3b–d). The all-*trans* enthalpy minimum of the alkanes gets deeper with increasing length, however, the increasing number of configurations available for intermediate separations gives rise to a concomitant rise in the entropic penalty for adopting the all-*trans* conformation. Interestingly, a secondary minimum in the enthalpy at methyl unit contact arises with increasing chain length. This secondary minimum is not as deep as the all-*trans* primary enthalpy minimum. Like the all-*trans* conformation, the secondary minimum is entropically unfavorable. Given that when the terminal methyl units are in contact the chain can adopt more conformations than those available in the all-*trans* conformation, this “ring closed” conformation is not as entropically unfavorable as the all-*trans* state. We may expect that for alkanes longer than C_20_ the chain may transition from an elongated conformation to a ring closed conformation. That chain length, however, is longer than the alkanes considered here. Nevertheless, the enthalpy reduction for ring closure with increasing chain length arising from attractive intramolecular van der Waals interactions underlies the stabilization of this state observed in vacuum (Figure 2a).

**Figure 4:**
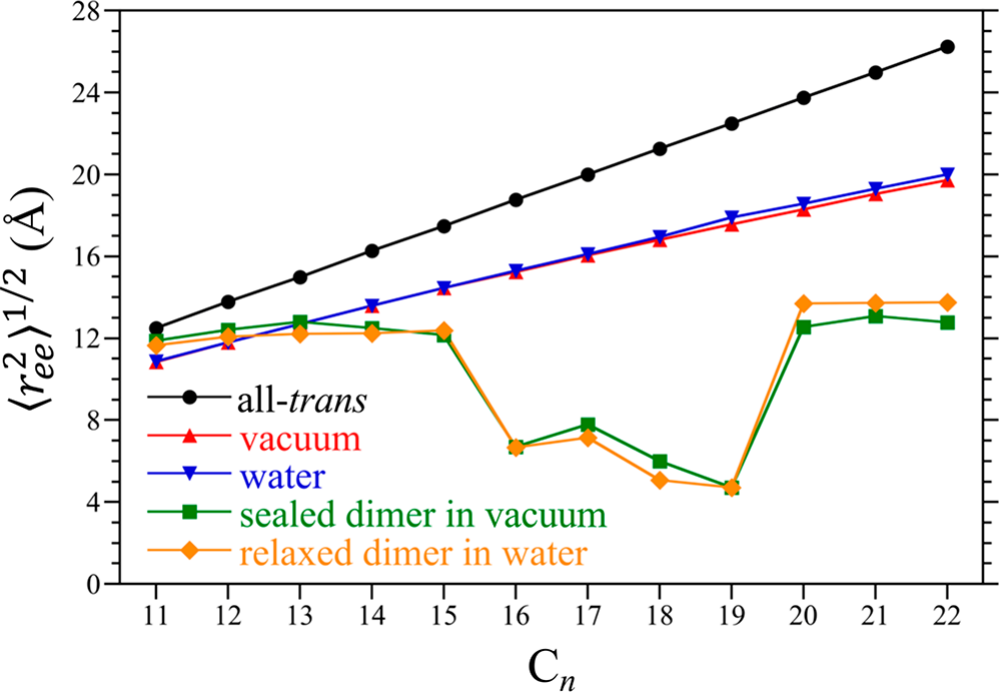
Root mean square end-to-end separations (⟨*r*_ee_^2^⟩^1/2^) of the alkanes (C_11_–C_22_) as a function of the number of carbons in the chain (*n*) at 25 °C. Results are reported for alkanes in vacuum, water, confined within the sealed cavitand dimer in vacuum, and within the unsealed dimer in water. In addition, we also include the end-to-end length of the alkanes if they adopted an all-*trans* conformation. The figure symbols are defined in the legend. Errors are comparable in size to the figure symbols.

The end-to-end PMFs for the alkanes in water are, to a first approximation, nearly the same as those in vacuum (Figure 2b). That is water does not appear to impact the overall conformation of the alkanes in difference to what would be expected if they underwent a hydrophobic collapse. Our results are in qualitative agreement with previous findings that alkanes on the order of 20 carbons in length and shorter do not collapse,^45^ although longer chains adopt globular conformations.^46^ The most significant difference between the alkane PMFs in water and vacuum is that the free energies of the ring closing state in water are more unfavorable by ∼*RT*. So at least up to C_22_ water appears to disfavor the ring closed state. Resultantly, the dominant conformations for the alkanes in vacuum and water are nearly indistinguishable. This can be more easily observed by considering the root-mean-square end-to-end separation, ⟨*r*_ee_^2^⟩^1/2^, of the alkanes (Figure 4). Notably, ⟨*r*_ee_^2^⟩^1/2^ for the alkanes in vacuum and water are effectively the same, so that we may conclude that the alkane conformations in water are largely dictated by intramolecular rather than solvent-mediated forces. Comparing ⟨*r*_ee_^2^⟩^1/2^ for the alkanes evaluated from simulation against that expected if the chains adopted an all-*trans* conformation, we find that the chains in vacuum and water are shorter than the all-*trans* chains. Moreover, the difference between the end-to-end length of the all-*trans* alkanes and ⟨*r*_ee_^2^⟩^1/2^ increases with increasing chain length. This is consistent with an increasing minority population of *gauche* dihedrals with increasing alkane length.

**Figure 5:**
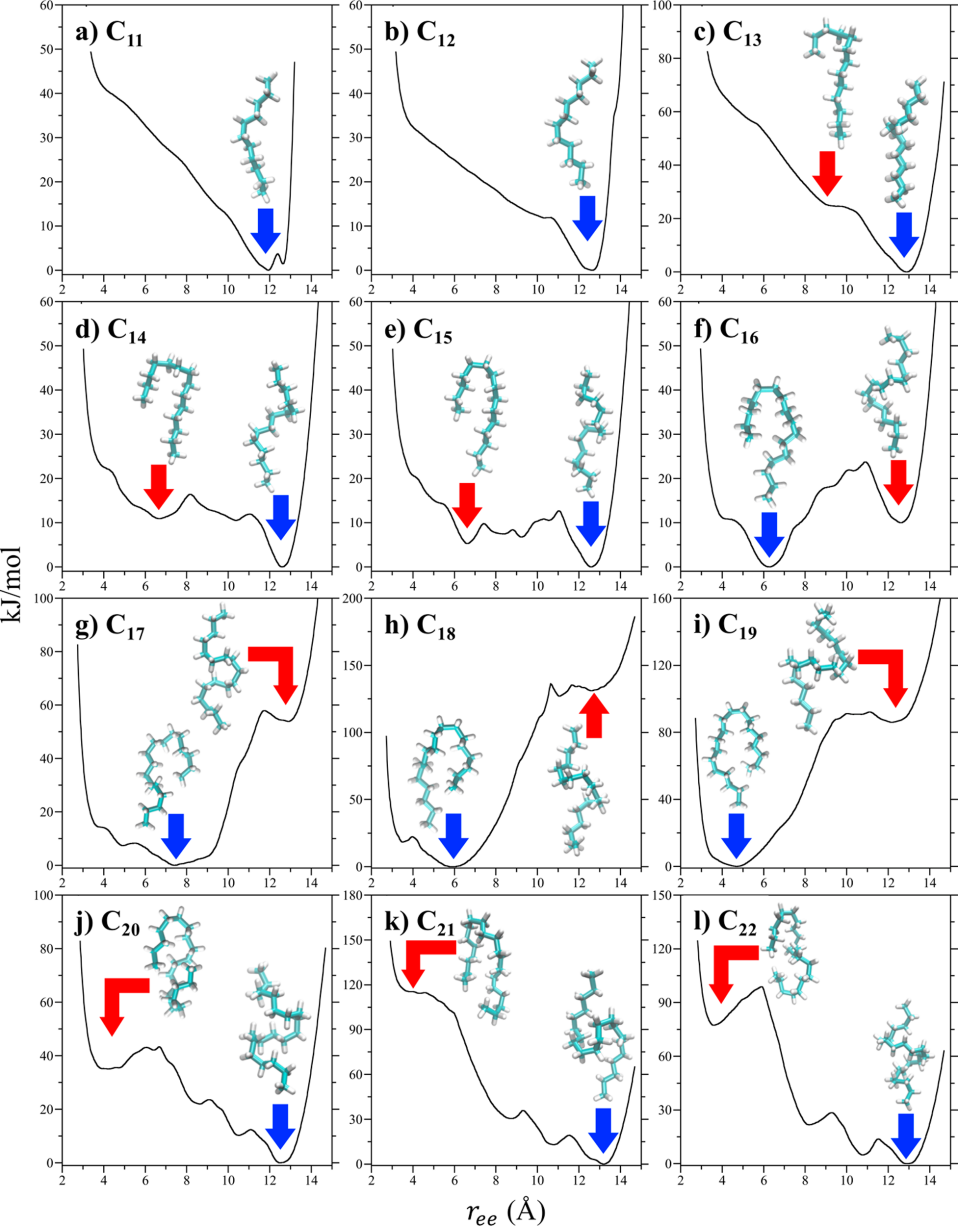
Potentials-of-mean force between the terminal methyl unit of the alkanes contained within sealed cavitand dimers in vacuum as a function of the end-to-end separation (*r*_ee_) between the terminal methyl units at 25 °C. The figures correspond to results for (**a**) C_11_, (**b**) C_12_, (**c**) C_13_, (**d**) C_14_, (**e**) C_15_, (**f**) C_16_, (**g**) C_17_, (**h**) C_18_, (**i**) C_19_, (**j**) C_20_, (**k**) C_21_, and (**l**) C_22_. The dominant chain conformations at the primary minimum (identified by the blue arrow) as determined following DPCA analysis are illustrated as the teal and white licorice structures for all alkanes. The dominant chain conformations at other prominent minima (identified by the red arrow) are also shown for alkanes C_13_–C_22_.

The conformational free energy landscapes of the alkanes confined within harmonically sealed OA dimer complexes in vacuum are distinct from those of the free chains in vacuum and water (Figure 5). Beginning with C_11_ (Figure 5a), the end-to-end PMF between its methyl units is qualitatively similar to that of the free chain in that it has a dominant primary minimum at ∼11.9 Å slightly shorter than an all-*trans* chain. The secondary minimum at 12.6 Å, however, has a free energy ∼*RT* lower than that of the free chain in vacuum. In addition, the shorter end-to-end separations to the left of the primary minimum are significantly more unfavorable under confinement than for the free chain. These observations indicate that under confinement, C_11_ favors extended conformations even more than the free chain does. Visual inspection of the dominant conformation of the chain at the primary minimum is consistent with an *extended* motif observed experimentally, although a slight bend near the middle of the chain is observed suggesting this conformation may not be readily distinguished from the *helical* motif. The interior of the dimer is akin to a prolate ellipsoid, wider in the middle and narrower at either end in the opposing cavitands. The bend likely arises from the chain attempting to maximize favorable van der Waals interactions by associating with the inner wall of the complex. Considering ⟨*r*_ee_^2^⟩^1/2^ for C_11_ under confinement (Figure 4), the alkane adopts a conformation longer than those of the free chains just shy of the all-*trans* chain.

The PMF profiles of C_12_ (Figure 5b) and C_13_ (Figure 5c) are similar to that of C_11_, exhibiting a primary minimum for an extended chain in the neighborhood of ∼12.7 Å. In difference to C_11_, however, we do not observe secondary minima for C_12_ or C_13_ corresponding to an all-*trans* chain. Given that the depth of an individual OA pocket is comparable to the length of a C_6_ chain, it stands to reason that the linear extent of the OA dimer’s interior volume is comparable to C_12_. As a result, it may not be possible for C_12_, let alone C_13_, to even adopt an all-*trans* conformation within the dimer. Visualizing the dominant conformational motifs of these chains at their primary minima, we categorize them as *extended*, although like C_11_, *gauche* dihedrals are observed. The root-mean-square end-to-end lengths of C_12_ and C_13_ (Figure 4) in this case are less than that of an all-*trans* chain and are comparable to that of C_11_ suggesting that the internal volume of the dimer is at most 13 Å in length. While the length of confined C_12_ is slightly greater than the free chain, the length of confined C_13_ is approximately equal to that of the free chain. Examining the PMF of C_13_ (Figure 5c) we also observe the potential initiation of a secondary minimum at end-to-end separations just to the left of the primary minimum, suggesting the potential for a second stable chain conformation for the confined guest with increasing length. Indeed, at the emerging secondary minimum of C_13_ the chain appears to adopt a *hairpin* motif.

For C_14_ (Figure 5d) the secondary minimum inferred from the PMFs of the shorter chains is more apparent. Specifically, we observe a dominant primary minimum at an end-to-end methyl separation of 12.6 Å and a secondary minimum near ∼7 Å. Visual inspection of the dominant conformation at the primary free energy minimum finds that instead of being *extended* the chain incorporates a conformational twist consistent with assignment of a *helical* motif. This free energy minimum, however, does not appear to be distinct from that associated with the *extended* motif assigned for the shorter alkanes. Rather, the chain adopts a helical twist so that the alkane fits within the dimeric complex. At the secondary minimum, the dominant conformation corresponds to a *hairpin* motif with the two methyl units directed toward one cavitand in the dimer and a turn directed toward the opposing cavitand. The free energy of the secondary minimum, however, is ∼10 kJ/mol greater than the primary minimum. As a result, the chain is expected to adopt a predominantly *helical* motif with brief excursions to a *hairpin* motif. Based on ⟨*r*_ee_^2^⟩^1/2^ the length of C_14_ is practically the same as that for C_11_–C_13_ and shorter than that of the free chain (Figure 4), reflecting an increase in the population of *gauche* dihedrals to form the helical twist to fit the guest within the capsule.

The end-to-end PMF profile of C_15_ (Figure 5e) is qualitatively similar to that of C_14_, although the free energy difference between the *helical* motif at 12.6 Å and *hairpin* motif at 6.6 Å drops from 10 to 5 kJ/mol. The *hairpin* becomes the dominant motif for C_16_ when we observe that the primary minimum in the PMF switches from 12.6 to 6.3 Å (Figure 5f). The *helical* conformer in this case is 10 kJ/mol less stable than the *hairpin*. The transition between the *helical* and *hairpin* free energy basins is accompanied by a drop in ⟨*r*_ee_^2^⟩^1/2^ from 12.2 to 6.7 Å (Figure 4). This transition from one conformational motif to another under confinement is reminiscent of a two-state transition in protein folding. Different from protein folding, however, the relative change in stability of the two free energy minima is not driven by changes in temperature but through changes in the length of the confined guest. Depending on the relative contributions of the enthalpy and entropy (*vide infra*), the observed motifs may be temperature dependent or independent.

The *hairpin* motif continues to dominate the free energy landscapes of C_17_ and C_18_ (Figures 5g and h). At the same time, the extended chain basin becomes increasingly unstable rising from 10 kJ/mol higher in free energy than the hairpin for C_16_ (Figure 5f) to 130 kJ/mol for C_18_ (Figure 5h). Interestingly, visualizing C_17_ and C_18_ at their secondary minimum near 12.6 Å, we find the *gauche* dihedrals of these alkane appear to migrate to the middle of the chains. This suggests the *helical* motif may be evolving into a fourth motif. Given that only *r*_ee_ is being used to quantify the conformational transition, however, we cannot assess whether this emerging motif is thermodynamically distinct from the *helical* motif. Increasing the alkane length to C_19_, we find that the free energy basin at 12.6 Å increases in stability with its free energy dropping from 130 kJ/mol for C_18_ to 85 kJ/mol for C_19_ relative to the *hairpin* (Figure 5i), suggesting the potential for a fourth conformational motif to emerge. The end-to-end lengths of C_16_–C_19_ are comparable to one another consistent with all of these chains adopting a *hairpin* motif (Figure 4).

Increasing the alkane length to C_20_, the dominant motif transitions once again (Figure 5j), with the free energy minimum shifting back to 12.5 Å. This conformational motif, however, appears to be distinct from the *helical* motif the alkanes previously adopted at this end-to-end length. Specifically, the twist for C_20_ now appears wider than the helices observed for C_14_ and C_15_. In difference to the *spinning top* motif (Figure 1b), however, the harmonically sealed dimer does not permit the turns to herniate between the two cavitands and partially expose the chain to the bulk. C_20_ subsequently appears to adopt two hairpin turns along its backbone that we refer to as the *chicane* motif. Below we examine the impact of releasing the dimer restraints for the complex in aqueous solution. In difference to the PMF landscapes of the shorter chains, we observe three additional progressively less stable minima in the end-to-end PMF of C_20_ at 10.5, 8.6, and 4.2 Å (Figure 5j). While the chain in the 4 Å basin appears to adopt a *hairpin*-like conformation, the alkane actually includes two turns such than the chain looks more like the letter “C”. These observations suggest the transition between the *hairpin* and *chicane* motifs is distinct and more complex than that between the *helical* and *hairpin* motifs discussed above. The transition between C_19_ and C_20_ is accompanied by a discrete change in ⟨*r*_ee_^2^⟩^1/2^, jumping from 4.7 to12.6 Å (Figure 4). The interior volume of the harmonically sealed cavitand dimer is 740 Å^3^ while the van der Waals volume of C_20_ is 350 Å^3^. The resultant packing fraction of the alkane within the dimer is 0.47, which is comparable to the packing fraction at which hard spheres freeze (0.49^47,48^). We surmise then that C_20_ and longer chains will experience significant packing frustration within the sealed dimers. The *chicane* motif remains the dominant conformation of C_21_ and C_22_ (Figure 5k and l). In qualitative agreement with C_20_, three progressively less stable minima in the end-to-end PMF are observed at shorter end-to-end separations than the primary minimum for C_21_ and C_22_. The value of ⟨*r*_ee_^2^⟩^1/2^ for C_21_ and C_22_ is nearly constant and ∼12.6 Å (Figure 4), equal to that of C_20_. This points to the fact that sealing the complexes in vacuum limits the ability of the alkanes to crack open the dimer to relieve packing frustration despite the fact the guest packing fractions of C_21_ and C_22_ within the complexes are 0.49 and 0.51, respectively.

**Figure 6:**
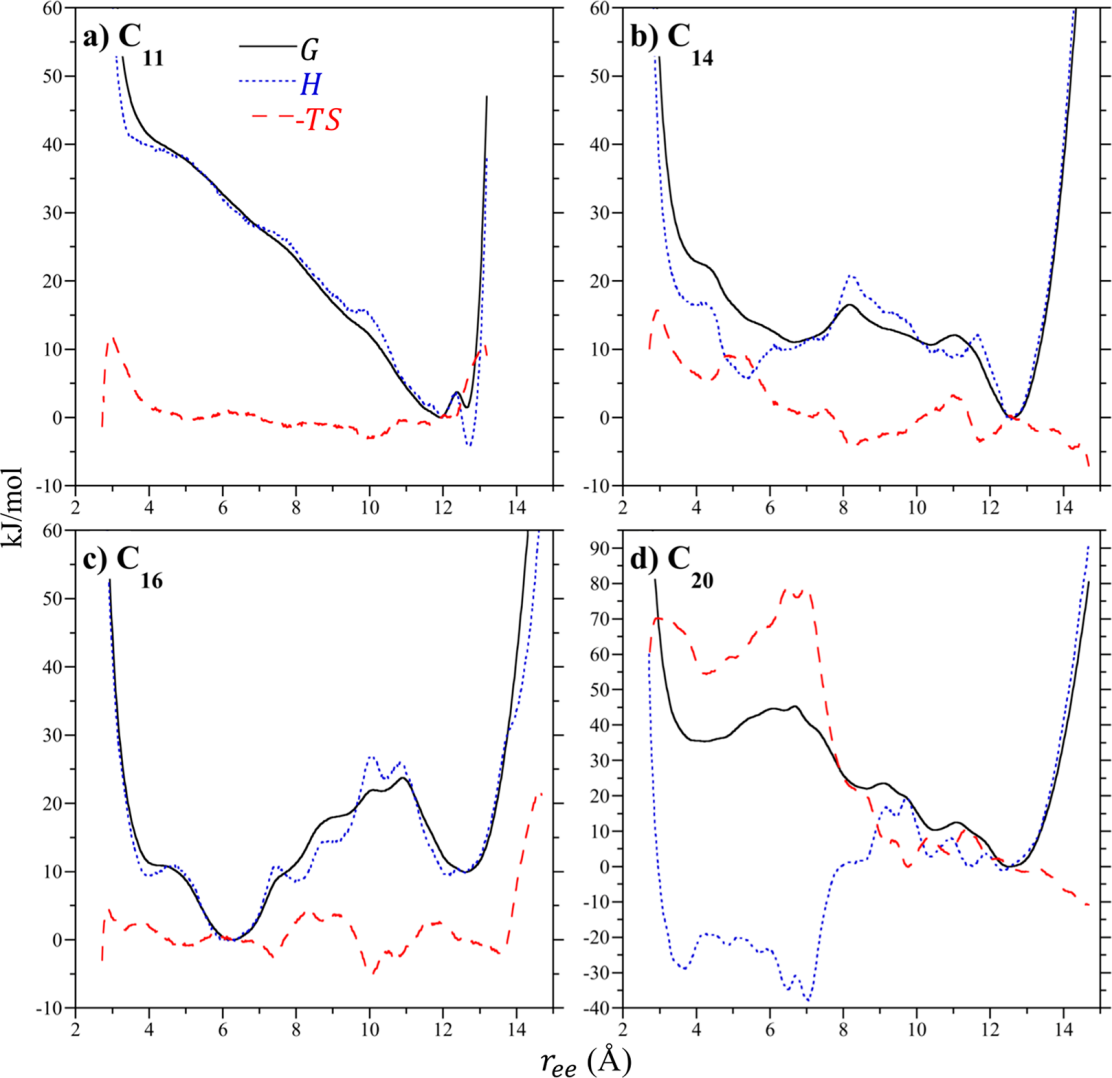
Decomposition of the alkane terminal methyl unit potentials of mean force into their enthalpic and entropic components for alkanes sealed in a dimer in vacuum at 25 °C. Results as reported for (**a**) C_11_, (**b**) C_14_, (**c**) C_16_, and (**d**) C_20_. These four chains are representative of the succession of conformational motifs from *extended*, to *helical*, to *hairpin*, and to *chicane*. The lines for the enthalpic (*H*) and entropic (−*TS*) contributions to the free energy/PMF (*G*) are defined in the legend of **a**.

Representative end-to-end PMFs of C_11_, C_14_, C_16_, and C_20_ within the sealed dimers are broken down into their enthalpic and entropic contributions at 25 °C in Figure 6. These alkanes were chosen to be representative of the *extended*, *helical*, *hairpin*, or *chicane* conformational motifs. In the case of C_11_ (Figure 6a), the PMF is dictated almost entirely by the end-to-end enthalpy. While the enthalpy favors the all-*trans* conformation, this conformation is disfavored by the entropy similar to that observed for the free chains in vacuum (Figure 3a). At shorter end-to-end separations, however, the entropy of the confined chain is nearly zero so that the enthalpy dominates. The enthalpy of the confined chain rises faster than that of the free chain with decreasing end-to-end length. We conclude then that confinement significantly limits the conformations available to C_11_ compared to the free chains. Moreover, the dimer presents an energetic confinement as suggested by the dominance of the enthalpy. Given the negligible role of the entropy in C_11_’s PMF, we expect over a reasonable temperature range (i.e., temperatures less than 500 K) that the *extended* motif is stable, consistent with the results of our REMD simulations at elevated temperatures.

The end-to-end enthalpy of C_14_ within the sealed dimer exhibits two minima associated with the *helical* (*r*_ee_ = 12.6 Å) and *hairpin* (*r*_ee_ = 5.3 Å) motifs (Figure 6b). The *helical* basin is largely controlled by the enthalpy while the end-to-end entropy near this free energy minimum is negligible and close to zero. The entropy of the *hairpin* basin is comparatively unfavorable and plays a minor role in shifting the free energy minimum from the local minimum in the enthalpy to slightly longer chain lengths. Nevertheless, extrapolating the temperature to absolute zero, while the *hairpin* motif will become more stable it will not overcome the stability of the *helical* motif. As with C_11_ then, we do not anticipate significant changes in the dominant conformation of C_14_ over a reasonable temperature range. The observation of a secondary minimum in the enthalpy at shorter end-to-end separations is qualitatively similar to what was observed for the free chain in vacuum (Figure 3b). The secondary enthalpy minimum under confinement, however, is enthalpically more favorable compared to the primary minimum than what was observed in vacuum, again pointing to a significant role for confinement to alter the enthalpic landscape of the confined chain.

The end-to-end enthalpy of C_16_ dominates its free energy landscape, controlling the shape and relative stability of the *hairpin* and *helical* basins at 6.2 and 12.6 Å (Figure 6c), respectively. The entropy plays a negligible role in determining the dominant chain conformation. As such, while the *hairpin* motif is expected to be dominant, the *helical* basin will be visited more frequently with increasing temperature. While the end-to-end enthalpy of the free chain does exhibit a minimum at shorter terminal methyl separations (Figure 3c), it is not the primary minimum in the enthalpy. Moreover, the entropy plays a more significant role in determining the free energy profile of the free chain than for the confined chain, for which the entropy plays almost no role.

In difference to the other guests considered, the end-to-end enthalpy and entropy of C_20_ confined within the sealed dimer plays a significant role in determining the observed guest conformation (Figure 6d). Notably, while the free energy favors the *chicane* motif (*r*_ee_ = 12.5 Å) the enthalpy exhibits a broad minimum from 3 to 7 Å associated with collapsed, ring closing conformations. These conformations, however, are more strongly opposed by an unfavorable entropy. As such, it might be expected that with diminishing temperature the collapsed states could dominate the observed conformer population. Given the magnitude of the product of the temperature and entropy, however, the potential for stabilizing these conformers lie below the freezing point of water and would subsequently not be observed experimentally. The disparity between the enthalpy and entropy of C_20_ and C_11_, C_14_, and C_16_ (Figures 6a–c) strongly suggests the transition to the *chicane* motif is distinct from that between the other motifs. Notably the large entropic penalty for C_20_ to adopt shorter conformations (*r*_ee_ < 7 Å) suggests that the frustration associated with trying to insert the two methyl units into a single cavitand results from the chain being only able to adopt limited conformations. This packing frustration is alleviated by redirecting the two terminal methyl units toward the two opposing host pockets so that the two turns settle near the wider middle of the dimer’s internal volume (Figure 5j) to enjoy more configurational freedom.

**Figure 7:**
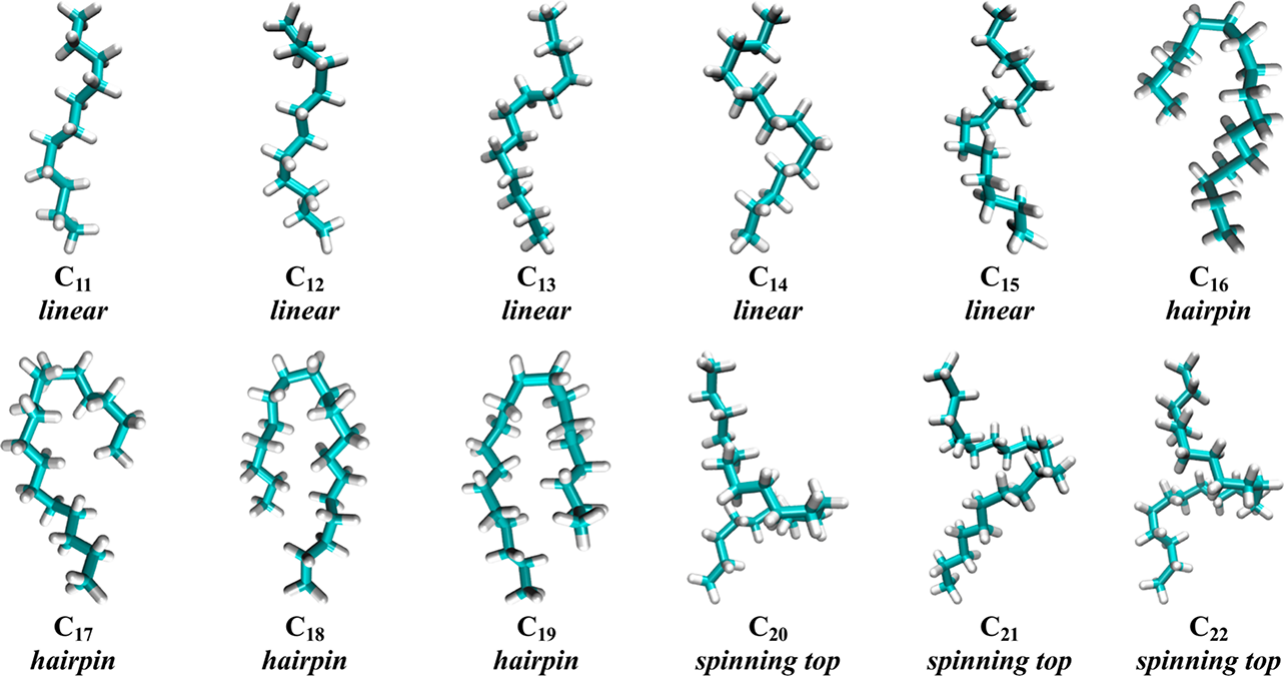
Relaxed alkane (C_11_–C_22_) guest conformations in unsealed cavitand dimers in water. The dominant alkane conformation was determined following DPCA analysis. The alkanes are illustrated in teal (carbon) and white (hydrogen) licorice format. In these snapshots the distinction between the *extended* and *helical* motifs (C_11_–C_15_) is subjective, although the degree of helicity appears to increase with the chain length. This highlights the conclusion that the *extended* and *helical* motifs do not appear to be thermodynamically distinct (Figure 5) but are members of a larger “*linear*” motif family. The alkanes are subsequently identified as belonging to either a *linear*, *hairpin*, or *spinning top* motif below each snapshot.

The *chicane* motif of C_20_–C_22_ found within the sealed dimers is not observed experimentally. While we believe the free energy landscapes of the guests within the sealed dimers in vacuum mirrors what would be observed for guests in an unsealed dimer in water, the imposed restraints limit breathing of the dimer complex that allow for separation of the two hosts and partial herniation of the guests. To assess the impact of unsealing the dimer on the bound alkane conformation, we harvested simulation configurations from the primary minima of each of the guests, placed the complex in aqueous solution, released the harmonic restraints, and simulated the guests within the unsealed complex for sufficient time to allow the chains to relax to their final equilibrium conformation. Snapshots of the dominant alkane guest conformations as determined from DPCA over these simulations are plotted in Figure 7. The equilibrated conformations of C_11_–C_19_ that adopt either *extended*/*helical* or *hairpin* motifs in the sealed dimers (Figures 5a–i) remain in that motif in the unsealed dimers. The chains exhibiting the *chicane* motif within the sealed dimers (C_20_–C_22_), however, relax to a *spinning top* motif within the unsealed dimers (Figure 7). In this case the turns migrate to the middle of the complex, merge, and are extruded between the partially separated hosts (Figure 1b). The greatest difference between ⟨*r*_ee_^2^⟩^1/2^ in the sealed and unsealed dimers occurs for C_20_–C_22_, for which the end-to-end separation grows by ∼1 Å as a result of the extrusion of alkane turn between the two cavitands in the unsealed dimer (Figure 4). The coincidence between the *spinning top* and *chicane* suggests that their stability has a common thermodynamic origin. The ability for the chains to relax from the *chicane* to *spinning top* morphology, however, indicates the free energy of the *spinning top* may be even lower than inferred from the sealed dimer calculations as a result of the chain lowering its packing fraction by partially opening the capsule.

Finally, we suggested in our initial simulation study of alkane conformations in cavitand dimers that the evolution from the *extended* and *helical* motifs does not correspond to a thermodynamic transition.^21^ The main difference between the sequence from C_11_ to C_15_ in the unsealed dimers in solution appears to be only a question of the extent of *gauche* dihedrals down the backbone for the chain to adopt a helical twist (Figure 7). As found in Figure 5, it is impossible for the longer alkanes to adopt an all-*trans* conformation and the population of *gauche* dihedrals must increase with increasing chain length so that the guest will even fit within the complex. The assignment of a *helical* motif is subsequently only a result of visual inspection of the dominant chain conformation. Indeed, comparing the dominant conformations of C_12_ and C_13_ in Figures 5 and 7 suggests an increase in the helical nature of the chains upon releasing the harmonic constraints. In addition, no clear differences in the primary minima of C_11_–C_15_ is found (Figure 5b–e). These observations provide deeper support for our previous proposition that *extended* and *helical* motifs are members of a larger *linear* motif family. The equilibrium between distinct free energy basins for the *linear* (*extended*/*helical*) to *hairpin* transition and *hairpin* to *chicane*/*spinning top* transition, however, indicates that these motifs are thermodynamically distinct. We subsequently identify the dominant guest conformers within the unsealed host dimers in water as belonging to the *linear*, *hairpin*, and *spinning top* motifs in Figure 7.

## Conclusions

While molecular simulation of free alkanes up to C_22_ in vacuum and aqueous solution do not exhibit conformational transitions, when these guests are confined within dimeric supramolecular complexes composed of cavitand hosts, a succession of conformational motifs from *extended*, to *helical*, to *hairpin*, to *chicane*/*spinning top* are observed. The *extended* and *helical* motifs do not appear to be thermodynamically distinct but fall within a larger *linear* motif family, visually distinguished only by the fraction of *gauche* dihedrals down the guest backbone forming a helical twist. The transition between the *linear*, *hairpin*, and *chicane*/*spinning top* motifs, however, appears to be thermodynamically distinct, with the transitions between the *linear* and *hairpin* motifs and *hairpin* and *chicane* motifs associated with jumps between free energy basins in the PMF profiles of the confined guests. Different from typical thermodynamic transitions driven by temperature changes, the conformational transitions under confinement are triggered by changes in the guest length. Specifically, examination of the PMF landscapes of the guests confined within harmonically sealed dimers found that the relative depths of primary and secondary minima in the free energy change systematically with increasing alkane length. For example, beginning with C_11_, the *hairpin*’s free energy basin, occurring at a shorter end-to-end distance than the *linear* basin, became increasingly stable with increasing chain length until it became the lowest free energy state beginning with C_16_. Similarly, the *chicane* free energy basin did not become dominant until C_20_, leading to a transition from the *hairpin* to *chicane* motifs. Examination of the thermodynamic components of the free energy found that confinement induces significant changes in the enthalpic and entropic landscape. Notably, the end-to-end entropy of free alkanes in vacuum was found to favor alkanes adopting conformations intermediate between ring closing and a fully extended chain. Confinement was found to significantly limit the conformations available, on the other hand, reducing the entropic penalties associated with adopting either ring closed or extended conformations for alkanes adopting the *linear* or *hairpin* motifs. As such, the end-to-end enthalpy plays a dominant role in determining the equilibrium conformation observed. In the case of the *chicane* motif, however, the entropy was found to play a dominating role in pushing the chain out of the *hairpin* basin and into the *chicane* basin despite the fact the enthalpy favors *hairpin*-like conformations. While the *chicane* motif is stable for chains C_20_ and longer within a harmonically sealed cavitand dimer, it is not observed experimentally. We attribute this to the fact that sealing the dimers does not permit the guests to potentially crack the complex open and partially expose themselves to the surrounding media. When we removed the harmonic restraints and equilibrated the dimer complexes in aqueous solution, we found the chains adopting the *chicane* relaxed to a *spinning top* motif with the alkanes partially herniating between the two hosts. The guests shorter than C_20_, however, retained the conformations observed in the sealed dimers in vacuum in the unsealed dimers in solution. This is attributable to the fact that alkanes C_20_ and longer exhibit packing fractions comparable to that of hard spheres near their freezing point, suggesting considerable packing frustration within the capsules that can be alleviated by pushing the hosts apart. Our simulations illustrate that confinement of alkanes significantly alters the free energy landscapes of guests bound within supramolecular complexes that can force the chains to adopt conformations that are not readily observed for unconfined, free chains. This, in turn, is reminiscent of the role of chaperones like the GroEL/GroES complex in refolding misfolded proteins by encapsulation within their internal volumes.

## References

[ref1] Mayhew M.; daSilva A. C. R.; Martin J.; ErdjumentBromage H.; Tempst P.; Hartl F. U. Protein Folding in the Central Cavity of the Groel-Groes Chaperonin Complex,. Nature 1996, 379, 420–426. 10.1038/379420a0. 8559246

[ref2] Xu Z. H.; Horwich A. L.; Sigler P. B. The Crystal Structure of the Asymmetric Groel-Groes-(Adp)(7) Chaperonin Complex. Nature 1997, 388, 741–750. 10.1038/41944. 9285585

[ref3] Munoz I. G.; Yebenes H.; Zhou M.; Mesa P.; Serna M.; Park A. Y.; Bragado-Nilsson E.; Beloso A.; de Carcer G.; Malumbres M.; et al. Crystal Structure of the Open Conformation of the Mammalian Chaperonin Cct in Complex with Tubulin. Nat. Struct. Mol. Biol. 2011, 18, 14–19. 10.1038/nsmb.1971. 21151115

[ref4] Gestaut D.; Limatola A.; Joachimiak L.; Frydman J. The Atp-Powered Gymnastics of Tric/Cct: An Asymmetric Protein Folding Machine with a Symmetric Origin Story. Curr. Opin. Struct. Biol. 2019, 55, 50–58. 10.1016/j.sbi.2019.03.002. 30978594 PMC6776438

[ref5] Zang Y. X.; Jin M. L.; Wang H. P.; Cui Z. C.; Kong L. L.; Liu C. X.; Cong Y. Staggered Atp Binding Mechanism of Eukaryotic Chaperonin Tric (Cct) Revealed through High-Resolution Cryo-Em. Nat. Struct. Mol. Biol. 2016, 23, 1083–1091. 10.1038/nsmb.3309. 27775711

[ref6] Purohit P. K.; Inamdar M. M.; Grayson P. D.; Squires T. M.; Kondev J.; Phillips R. Forces During Bacteriophage DNA Packaging and Ejection. Biophys. J. 2005, 88, 851–866. 10.1529/biophysj.104.047134. 15556983 PMC1305160

[ref7] Purohit P. K.; Kondev J.; Phillips R. Mechanics of DNA Packaging in Viruses. Proc. Natl. Acad. Sci. U. S. A. 2003, 100, 3173–3178. 10.1073/pnas.0737893100. 12629206 PMC404299

[ref8] Grayson P.; Evilevitch A.; Inamdar M. M.; Purohit P. K.; Gelbart W. M.; Knobler C. M.; Phillips R. The Effect of Genome Length on Ejection Forces in Bacteriophage Lambda. Virology 2006, 348, 430–436. 10.1016/j.virol.2006.01.003. 16469346 PMC3178461

[ref9] Jordan J. H.; Gibb B. C. Molecular Containers Assembled through the Hydrophobic Effect. Chem. Soc. Rev. 2015, 44, 547–585. 10.1039/C4CS00191E. 25088697

[ref10] Gibb C. L. D.; Gibb B. C. Well-Defined, Organic Nanoenvironments in Water: The Hydrophobic Effect Drives a Capsular Assembly. J. Am. Chem. Soc. 2004, 126, 11408–11409. 10.1021/ja0475611. 15366865

[ref11] Gan H. Y.; Benjamin C. J.; Gibb B. C. Nonmonotonic Assembly of a Deep-Cavity Cavitand. J. Am. Chem. Soc. 2011, 133, 4770–4773. 10.1021/ja200633d. 21401093

[ref12] Tang D.; Barnett J. W.; Gibb B. C.; Ashbaugh H. S. Guest Controlled Nonmonotonic Deep Cavity Cavitand Assembly State Switching. J. Phys. Chem. B 2017, 121, 10717–10725. 10.1021/acs.jpcb.7b09021. 29099596

[ref13] Gibb C. L. D.; Gibb B. C. Straight-Chain Alkanes Template the Assembly of Water-Soluble Nano-Capsules. Chem. Commun. 2007, 1635–1637. 10.1039/b618731e. 17530084

[ref14] Liu S. M.; Russell D. H.; Zinnel N. F.; Gibb B. C. Guest Packing Motifs within a Supramolecular Nanocapsule and a Covalent Analogue. J. Am. Chem. Soc. 2013, 135, 4314–4324. 10.1021/ja310741q. 23448338 PMC3613047

[ref15] Choudhury R.; Barman A.; Prabhakar R.; Ramamurthy V. Hydrocarbons Depending on the Chain Length and Head Group Adopt Different Conformations within a Water-Soluble Nanocapsule: H-1 Nmr and Molecular Dynamics Studies. J. Phys. Chem. B 2013, 117, 398–407. 10.1021/jp3090815. 23215251

[ref16] Gibb C. L. D.; Sundaresan A. K.; Ramamurthy V.; Gibb B. C. Templation of the Excited-State Chemistry of (Alpha-(N-Alkyl) Dibenzyl Ketones: How Guest Packing within a Nanoscale Supramolecular Capsule Influences Photochemistry. J. Am. Chem. Soc. 2008, 130, 4069–4080. 10.1021/ja7107917. 18321108

[ref17] Wang K. Y.; Cai X. Y.; Yao W.; Tang D.; Kataria R.; Ashbaugh H. S.; Byers L. D.; Gibb B. C. Electrostatic Control of Macrocyclization Reactions within Nanospaces. J. Am. Chem. Soc. 2019, 141, 6740–6747. 10.1021/jacs.9b02287. 30929421

[ref18] Cai X. Y.; Kataria R.; Gibb B. C. Intrinsic and Extrinsic Control of the Pk(a) of Thiol Guests inside Yoctoliter Containers. J. Am. Chem. Soc. 2020, 142, 8291–8298. 10.1021/jacs.0c00907. 32271561

[ref19] Yu X. C.; Tang W. Q.; Zhao T.; Jin Z. H.; Zhao S. L.; Liu H. L. Confinement Effect on Molecular Conformation of Alkanes in Water-Filled Cavitands: A Combined Quantum/Classical Density Functional Theory Study. Langmuir 2018, 34, 13491–13496. 10.1021/acs.langmuir.8b02209. 30350710

[ref20] Ashbaugh H. S.; Gibb B. C.; Suating P. Cavitand Complexes in Aqueous Solution: Collaborative Experimental and Computational Studies of the Wetting, Assembly, and Function of Nanoscopic Bowls in Water. J. Phys. Chem. B 2021, 125, 3253–3268. 10.1021/acs.jpcb.0c11017. 33651614 PMC8040017

[ref21] Barnett J. W.; Gibb B. C.; Ashbaugh H. S. Succession of Alkane Conformational Motifs Bound within Hydrophobic Supramolecular Capsular Assemblies. J. Phys. Chem. B 2016, 120, 10394–10402. 10.1021/acs.jpcb.6b06496. 27603416

[ref22] García A. E. Large-Amplitude Nonlinear Motions in Proteins. Phys. Rev. Lett. 1992, 68, 2696–2699. 10.1103/PhysRevLett.68.2696. 10045464

[ref23] Altis A.; Nguyen P. H.; Hegger R.; Stock G. Dihedral Angle Principal Component Analysis of Molecular Dynamics Simulations. J. Chem. Phys. 2007, 126, 244111 10.1063/1.2746330. 17614541

[ref24] Altis A.; Otten M.; Nguyen P. H.; Hegger R.; Stock G. Construction of the Free Energy Landscape of Biomolecules Via Dihedral Angle Principal Component Analysis. J. Chem. Phys. 2008, 128, 245102 10.1063/1.2945165. 18601386

[ref25] Amadei A.; Linssen A. B. M.; Berendsen H. J. C. Essential Dynamics of Proteins. Proteins: Struct., Funct., Genet. 1993, 17, 412–425. 10.1002/prot.340170408. 8108382

[ref26] Mu Y. G.; Nguyen P. H.; Stock G. Energy Landscape of a Small Peptide Revealed by Dihedral Angle Principal Component Analysis. Proteins: Struct., Funct., Genet. 2005, 58, 45–52. 10.1002/prot.20310. 15521057

[ref27] Ditchfield R. Self-Consistent Perturbation-Theory of Diamagnetism. 1. Gauge-Invariant Lcao Method for Nmr Chemical-Shifts. Mol. Phys. 1974, 27, 789–807. 10.1080/00268977400100711.

[ref28] Abraham M. J.; Murtola T.; Schulz R.; Páll S.; Smith J. C.; Hess B.; Lindahl E. Gromacs: High Performance Molecular Simulations through Multi-Level Parallelism from Laptops to Supercomputers. SoftwareX 2015, 1–2, 19–25. 10.1016/j.softx.2015.06.001.

[ref29] Siu S. W. I.; Pluhackova K.; Bockmann R. A. Optimization of the Opls-Aa Force Field for Long Hydrocarbons. J. Chem. Theory Comput. 2012, 8, 1459–1470. 10.1021/ct200908r. 26596756

[ref30] Wang J. M.; Wolf R. M.; Caldwell J. W.; Kollman P. A.; Case D. A. Development and Testing of a General Amber Force Field. J. Comput. Chem. 2004, 25, 1157–1174. 10.1002/jcc.20035. 15116359

[ref31] Jakalian A.; Jack D. B.; Bayly C. I. Fast, Efficient Generation of High-Quality Atomic Charges. Am1-Bcc Model: Ii. Parameterization and Validation. J. Comput. Chem. 2002, 23, 1623–1641. 10.1002/jcc.10128. 12395429

[ref32] Ewell J.; Gibb B. C.; Rick S. W. Water inside a Hydrophobic Cavitand Molecule. J. Phys. Chem. B 2008, 112, 10272–10279. 10.1021/jp804429n. 18661937

[ref33] Horn H. W.; Swope W. C.; Pitera J. W.; Madura J. D.; Dick T. J.; Hura G. L.; Head-Gordon T. Development of an Improved Four-Site Water Model for Biomolecular Simulations: Tip4p-Ew. J. Chem. Phys. 2004, 120, 9665–9678. 10.1063/1.1683075. 15267980

[ref34] Hess B.; Bekker H.; Berendsen H. J. C.; Fraaije J. Lincs: A Linear Constraint Solver for Molecular Simulations. J. Comput. Chem. 1997, 18, 1463–1472. 10.1002/(SICI)1096-987X(199709)18:12<1463::AID-JCC4>3.0.CO;2-H.

[ref35] Miyamoto S.; Kollman P. A. Settle - an Analytical Version of the Shake and Rattle Algorithm for Rigid Water Models. J. Comput. Chem. 1992, 13, 952–962. 10.1002/jcc.540130805.

[ref36] Nosé S. A Unified Formulation of the Constant Temperature Molecular-Dynamics Methods. J. Chem. Phys. 1984, 81, 511–519. 10.1063/1.447334.

[ref37] Hoover W. G. Canonical Dynamics: Equilibrium Phase-Space Distributions. Phys. Rev. A: At., Mol., Opt. Phys. 1985, 31, 1695–1697. 10.1103/PhysRevA.31.1695. 9895674

[ref38] Torrie G. M.; Valleau J. P. Non-Physical Sampling Distributions in Monte Carlo Free-Energy Estimation: Umbrella Sampling. J. Comput. Phys. 1977, 23, 187–199. 10.1016/0021-9991(77)90121-8.

[ref39] Kumar S.; Bouzida D.; Swendsen R. H.; Kollman P. A.; Rosenberg J. M. The Weighted Histogram Analysis Method for Free Energy Calculation on Biomolecules. 1. The Method. J. Comput. Chem. 1992, 13, 1011–1021. 10.1002/jcc.540130812.

[ref40] Bussi G.; Donadio D.; Parrinello M. Canonical Sampling through Velocity Rescaling. J. Chem. Phys. 2007, 126, 014101 10.1063/1.2408420. 17212484

[ref41] Patriksson A.; van der Spoel D. A Temperature Predictor for Parallel Tempering Simulations. Phys. Chem. Chem. Phys. 2008, 10, 2073–2077. 10.1039/b716554d. 18688361

[ref42] Parrinello M.; Rahman A. Polymophic Transitions in Single-Crystals - a New Molecular-Dynamics Method. J. Appl. Phys. 1981, 52, 7182–7190. 10.1063/1.328693.

[ref43] Darden T.; York D.; Pedersen L. Particle Mesh Ewald: An N. Log(N) Method for Ewald Sums in Large Systems. J. Chem. Phys. 1993, 98, 10089–10092. 10.1063/1.464397.

[ref44] Sugita Y.; Okamoto Y. Replica-Exchange Molecular Dynamics Method for Protein Folding. Chem. Phys. Lett. 1999, 314, 141–151. 10.1016/S0009-2614(99)01123-9.

[ref45] Ferguson A. L.; Debenedetti P. G.; Panagiotopoulos A. Z. Solubility and Molecular Conformations of N-Alkane Chains in Water. J. Phys. Chem. B 2009, 113, 6405–6414. 10.1021/jp811229q. 19361179

[ref46] Chakrabarty S.; Bagchi B. Self-Organization of N-Alkane Chains in Water: Length Dependent Crossover from Helix and Toroid to Molten Globule. J. Phys. Chem. B 2009, 113, 8446–8448. 10.1021/jp9034387. 19485315

[ref47] Hoover W. G.; Ree F. H. Melting Transition and Communal Entropy for Hard Spheres. J. Chem. Phys. 1968, 49, 3609–3617. 10.1063/1.1670641.

[ref48] Noya E. G.; Vega C.; de Miguel E. Determination of the Melting Point of Hard Spheres from Direct Coexistence Simulation Methods. J. Chem. Phys. 2008, 128, 154507 10.1063/1.2901172. 18433235

